# An Analysis of Pharmacogenomic-Guided Pathways and Their Effect on Medication Changes and Hospital Admissions: A Systematic Review and Meta-Analysis

**DOI:** 10.3389/fgene.2021.698148

**Published:** 2021-07-30

**Authors:** Victoria David, Beth Fylan, Eleanor Bryant, Heather Smith, Gurdeep S. Sagoo, Marcus Rattray

**Affiliations:** ^1^Leeds Teaching Hospitals National Health Service (NHS) Trust, Leeds, United Kingdom; ^2^School of Pharmacy and Medical Sciences, University of Bradford, Bradford, United Kingdom; ^3^Wolfson Centre for Applied Health Research, Bradford, United Kingdom; ^4^Yorkshire and Humber Patient Safety Translational Research Centre, Bradford Institute of Health Research, Bradford, United Kingdom; ^5^Division of Psychology in the School of Social Sciences, University of Bradford, Bradford, United Kingdom; ^6^Academic Unit of Health Economics, Leeds Institute of Health Sciences, University of Leeds, Leeds, United Kingdom; ^7^National Institute for Health Research Leeds *In Vitro* Diagnostics Co-operative, Leeds Teaching Hospitals NHS Trust, Leeds, United Kingdom

**Keywords:** pharmacogenetic testing, hospital admission, medication change, adverse drug reaction, patient care pathway, medicines optimization

## Abstract

Ninety-five percent of the population are estimated to carry at least one genetic variant that is discordant with at least one medication. Pharmacogenomic (PGx) testing has the potential to identify patients with genetic variants that puts them at risk of adverse drug reactions and sub-optimal therapy. Predicting a patient's response to medications could support the safe management of medications and reduce hospitalization. These benefits can only be realized if prescribing clinicians make the medication changes prompted by PGx test results. This review examines the current evidence on the impact PGx testing has on hospital admissions and whether it prompts medication changes. A systematic search was performed in three databases (Medline, CINAHL and EMBASE) to search all the relevant studies published up to the year 2020, comparing hospitalization rates and medication changes amongst PGx tested patients with patients receiving treatment-as-usual (TAU). Data extracted from full texts were narratively synthesized using a process model developed from the included studies, to derive themes associated to a suggested workflow for PGx-guided care and its expected benefit for medications optimization and hospitalization. A meta-analysis was undertaken on all the studies that report the number of PGx tested patients that had medication change(s) and the number of PGx tested patients that were hospitalized, compared to participants that received TAU. The search strategy identified 5 hospitalization themed studies and 5 medication change themed studies for analysis. The meta-analysis showed that medication changes occurred significantly more frequently in the PGx tested arm across 4 of 5 studies. Meta-analysis showed that all-cause hospitalization occurred significantly less frequently in the PGx tested arm than the TAU. The results show proof of concept for the use of PGx in prescribing that produces patient benefit. However, the review also highlights the opportunities and evidence gaps that are important when considering the introduction of PGx into health systems; namely patient involvement in PGx prescribing decisions, thus a better understanding of the perspective of patients and prescribers. We highlight the opportunities and evidence gaps that are important when considering the introduction of PGx into health systems.

## Introduction

Many individuals carry genetic variants that can have a profound impact on how they respond to medications (McInnes et al., 2021). Pharmacogenomics (PGx) is the use of genomic information to understand individual responses to medications. PGx promises a personalized approach to safe and effective management of health conditions and reducing the inappropriate use of multiple medications (inappropriate polypharmacy), by guiding the selection of appropriate medications (Sharp et al., 2019).

Genetic variants are described as being discordant with a medication when the genetic variant predisposes an individual to having an unfavorable response to medications. An unfavorable drug response can consist of an adverse drug reaction or poor drug efficacy (Wei et al., 2012). For example, patients with a variation of the CYP2D6 gene that makes them ultra-rapid metabolisers of the analgesic, codeine, may experience an adverse event such as respiratory depression which can prove fatal (Kirchheiner et al., 2007). An example of poor drug efficacy relates to people with loss-of-function variants of the gene CYP2C19 that make them poor metabolisers of clopidogrel so they are unable to derive therapeutic benefit from taking the medication (Brown and Pereira, 2018). The potential for unfavorable response to medications in the general population due to PGx variants is high, with 95% of the population estimated to carry one or more genetic variants that are discordant with at least one medication (Van Driest et al., 2014; Bush et al., 2016; Ji et al., 2016; Mostafa et al., 2019). Participants of a large study that analyzed PGx genetic variation in the UK Biobank, found that participants on average had discordant genetic variants to at least 10 medications (McInnes et al., 2021). A study in the USA found that 50% of patients with Medicare health insurance aged 65 and over, received at least one medication affected by genetic variability, sometimes referred to as a PGx medications, with 25–30% receiving at least two PGx medications (Mostafa et al., 2019). Discordant variants are potentially actionable, i.e., amenable to PGx-guided change in prescribing for an individual to optimize medication effectiveness and to reduce adverse effects.

Older people are more likely to have multiple health conditions (multimorbidity) and be prescribed multiple medications (polypharmacy) (Pedrós et al., 2014). Polypharmacy is associated with an increased risk of adverse drug reactions (Hanlon et al., 2018) and hospitalization (Alexopoulou et al., 2008; Leendertse et al., 2008; Pedrós et al., 2014). Pedrós et al. (2014) reports a 5 and 9-fold increased risk in hospitalizations related to adverse drug reactions amongst patients using more than three and 10 medications, respectively. In addition, people aged 65 and over constituted 76% of hospitalizations related to adverse drug reactions in the study (Pedrós et al., 2014). Age and polypharmacy are identified as independent risk factors of hospitalizations related to adverse drug reactions (Pedrós et al., 2014). Therefore, if 20–40% of variability to medication response between individuals is due to a patient's genetic profile (Ingelman-Sundberg, 2001), then the development of intervention strategies like PGx-guided therapy could aid the tailoring of dose and dosing regimen, or selection of medications, to a patient's genetic profile. Tailoring medication using PGx information, could reduce adverse drug reactions so that hospitalization related to adverse drug reactions is reduced, especially in older people who are more vulnerable to the consequences of inappropriate prescribing.

As institutions and healthcare systems look to begin adopting PGx-guided prescribing (Luzum et al., 2017; Alshabeeb et al., 2019; Turner et al., 2020), it is important to understand its potential for improving patient outcomes and its effect on prescribing practice. At the time of this writing, we are not aware of any review that considers the effectiveness of PGx testing on medication changes and hospital admissions. To construct a patient care pathway that incorporates PGx-guided pharmacotherapy, it is important to know whether a planned intervention results in changes to prescribing and improved patient outcomes. If PGx testing can lower medication-related hospital admissions, then PGx testing has the potential to improve medications safety and efficacy in patients, as well as potentially producing cost-savings for health care systems. The aim of this rapid review, therefore, is to synthesize the current evidence that associates PGx testing and unplanned hospital admissions and whether prescribers accept PGx-guided recommendations for changes to prescribed medication.

## Methods

This rapid review used systematic review methodology (Grant and Booth, 2009) and adheres to the Preferred Reporting Items for Systematic Review (PRISMA) with meta-analysis (Liberati et al., 2009).

### Criteria for Considering Studies

We used the PICOS (Population, Intervention, Comparator, Outcome, Settings) tool to identify relevant keywords/Medical Subject Headings (MeSH) words.

### Type of Participants

Any participants taking medication for a health condition.

### Type of Intervention and Comparator

PGx testing as an intervention compared to standard pharmacotherapy not guided by PGx.

### Type of Outcomes

Frequency of hospitalization and medication changes made by the prescriber.

### Study Settings

Studies were included with PGx-guided pharmacotherapy implemented in either primary or secondary care settings of a healthcare system, irrespective of specialization (e.g., psychiatry, cardiothoracic, general) or country.

### Search Strategy

Boolean operators AND/OR were used to combine search terms and the use of Medical Subject Headings (MeSH) helped to retrieve information from the relevant topic area regardless of the terms used by the authors to refer to the same concept or spelling.

Two literature searches were conducted discretely for the themes “hospital admission” and “medication change.” The literature search for the “hospital admission” theme was conducted using MEDLINE (EBSCOHost platform), CINAHL (EBSCOHost platform) and Embase. An information specialist was consulted to ensure an adequate balance of sensitivity and specificity in the search strategy used. We used the following databases to identify primary research studies for inclusion.

- MEDLINE 2000 to 2020, EBSCOHost (searched 10th January 2021).
- CINAHL 2016 to 2020, EBSCOHost (searched 10th January 2021).
- Embase 1996 to 2021, searched (searched 10th January 2021).

### Electronic Searches

Searches were conducted using the following sources below.

### Hospital Admission Themed Searches

Medline and CINAHL (EBSCOHost platform)—the keywords used were [(MH “Pharmacogenetics”) OR (MH “Pharmacogenomic Testing”) OR (MH “Pharmacogenomic Variants”)] AND [(MH “Patient Admission”) OR (MH “Patient Readmission”) OR (MH “Hospitalization”)].

Embase- the keywords used were [(“pharmacogenetic testing”/exp OR “pharmacogenetic analysis” OR “pharmacogenetic screening” OR “pharmacogenetic study” OR “pharmacogenetic testing” OR “pharmacogenomic analysis” OR “pharmacogenomic screening” OR “pharmacogenomic study” OR “pharmacogenomic testing” OR “genotyping”) AND (“hospital admission” OR “readmission”)].

### Medication Changes Themed Search

Medline (EBSCOHost platform)- the keywords used were [“test^*^” AND ((MH “Pharmacogenomic Testing”) OR “pharmacogenomic” OR (MH “Pharmacogenetics”) OR “pharmacogenetic^*^” OR (MH “Pharmacogenomic Variants) OR “variant^*^” OR “genotype^*^”))] AND (“medication change^*^” OR “medicine change^*^” OR [(“change” AND (“medication” OR “prescription” OR “drug”))].

We did not apply any limits on language or publication date. We searched all databases from inception to the date of search (10th January 2021).

The hospital admissions theme was the first literature search that was undertaken, and it became apparent that all the records selected for narrative synthesis could be found in the MEDLINE database and the records on MEDLINE were more relevant to the topic of study than the records from the other two databases used. As a result of this observation and the timely manner to which rapid reviews aim to achieve evidence synthesis, only MEDLINE (EBSCOHost platform) was used to conduct the literature search for the “medication change” theme. Clavirate analytics EndNote web was the citation manager used to store the search results which were de-duplicated both using EndNote and completed manually^1^.

### Study Selection

The eligibility criteria used for the selection of studies are based on the PICOS (Population, Intervention, Comparison, Outcome and Setting) framework. The included studies featured patients taking medication (population), pharmacogenomic testing as an intervention, compared to standard pharmacotherapy not guided by pharmacogenomics, with hospitalization or medication adjustments as an outcome. Experimental (i.e., RCTs) and observational studies set in primary/secondary facets of the healthcare system were included. This framework was used as a screening-and-selection tool for a two-stage screening and selection process. At the first stage, the title and abstract of each article was screened independently by one reviewer (VD), for relevancy to the area of pharmacogenomic testing, hospital admission and medication changes. For the hospital admission themed studies, a co-author (MR) double screened the title and abstract for 10% of the total number of articles. In the second step, the full texts for the selected studies were obtained and they were assessed for inclusion using the PICOS framework by VD.

### Quality Assessment Tool

The methodologically diverse nature of the research designs used across the eligible studies required three CASP checklists for the three eligible study designs (randomized control trial, case-control study and cohort study) be used to critically appraise the validity, trustworthiness and reliability of the studies. Three checklists were used as quality assessment tools:

- The new CASP critical appraisal tool for randomized controlled trials (RCTs) standard checklist that takes into account the widely adopted CONSORT 2010 guideline for reporting RCTs^2^.
- CASP critical appraisal tool for cohort study^3^.
- CASP critical appraisal tool for case control study^4^.

Information about study design was collected to help select an appropriate CASP (critical appraisal skills programme) appraisal tool and to assess study quality for each study so that the strength of the studies could be established. Information on age, specialty, setting, test, and genes tested will help to determine potential barriers and facilitators to implementing pharmacogenomic testing in routine clinical practice. The CASP checklists used in this rapid review, were modified to exclude non-relevant questions that ask the reviewer to consider the applicability of results to meet local needs.

### Data Extraction

Data extraction was independently carried out from five “hospital admission” themed studies and five “medication change” themed studies. The following information for all the studies was extracted: study characteristics (author, study design, number of participants, mean age of participants, specialty, setting, single-gene or panel test, pharmacokinetic/pharmacodynamic genes tested, whether a pharmacist featured in reviewing pharmacogenomic test results/recommendations, outcome variable and outcomes).

### Narrative Synthesis

A narrative synthesis of studies meeting the inclusion criteria, was conducted. Narrative synthesis involves combining the findings of multiple studies using a textual approach to create a summary and explain the findings from the included studies (Popay et al., 2006). The method of narrative synthesis is used when substantial methodological and clinical heterogeneity between studies render studies non-amenable to meta-analysis. However, a forest plot is used, to present results in a clear way.

The results section describes the inclusion/exclusion of studies, presents the data extracted from studies from both themes (hospital admission and medication changes) and a summary of the effectiveness results of PGx testing on hospital admissions and medication changes.

The quantative data from all studies for all-cause hospitalization was combined for meta-analysis, as presented in a forest plot and associated table.

### Data Analysis

A meta-analysis of the quantitative data on all-cause hospitalization and occurrence of medication changes was conducted for the hospitalization themed studies and medication change themed studies. The meta-analysis was performed using Review Manager 5.4.1^5^ and is presented in two separate forest plots for the hospitalization themed studies and medication change theme studies. Both forest plots used a random effect model for meta-analysis to account for heterogeneity between the included studies. All outcomes were handled as dichotomous variables and odds ratios with 95% confidence intervals (95% CI) were calculated.

Heterogeneity was tested with Chi^2^ and *I*^2^ tests. Thresholds for the interpretation of *I*^2^ values were interpreted as “might not be important” (0–40%), “moderate” (30–60%), “substantial” (50–90%), and “considerable” (75–100%) heterogeneity with a *p* < 0.1 considered significant, as suggested by the Cochrane Handbook (Deeks et al., 2021). Given the large heterogeneity that was observed, we undertook a Baujat plot analysis (Baujat et al., 2002) which is a diagnostic plot to detect studies which are believed to be excessively contributing to the heterogeneity of a meta-analysis. The plot shows the contribution to the overall heterogeneity of each study as measured by Cochran's Q*Q* (X-axis) vs. its influence on the pooled effect size on the Y-axis. Influence analyses proposed by Viechtbauer and Cheung (2010) was also conducted to identify the influential studies with extreme values in the graphs. Both the Baujat plot and the Influence analyses were performed in R language using the Harrer et al. (2021) handbook.

## Results

### Inclusion and Exclusion of Studies

**Figure 1:**
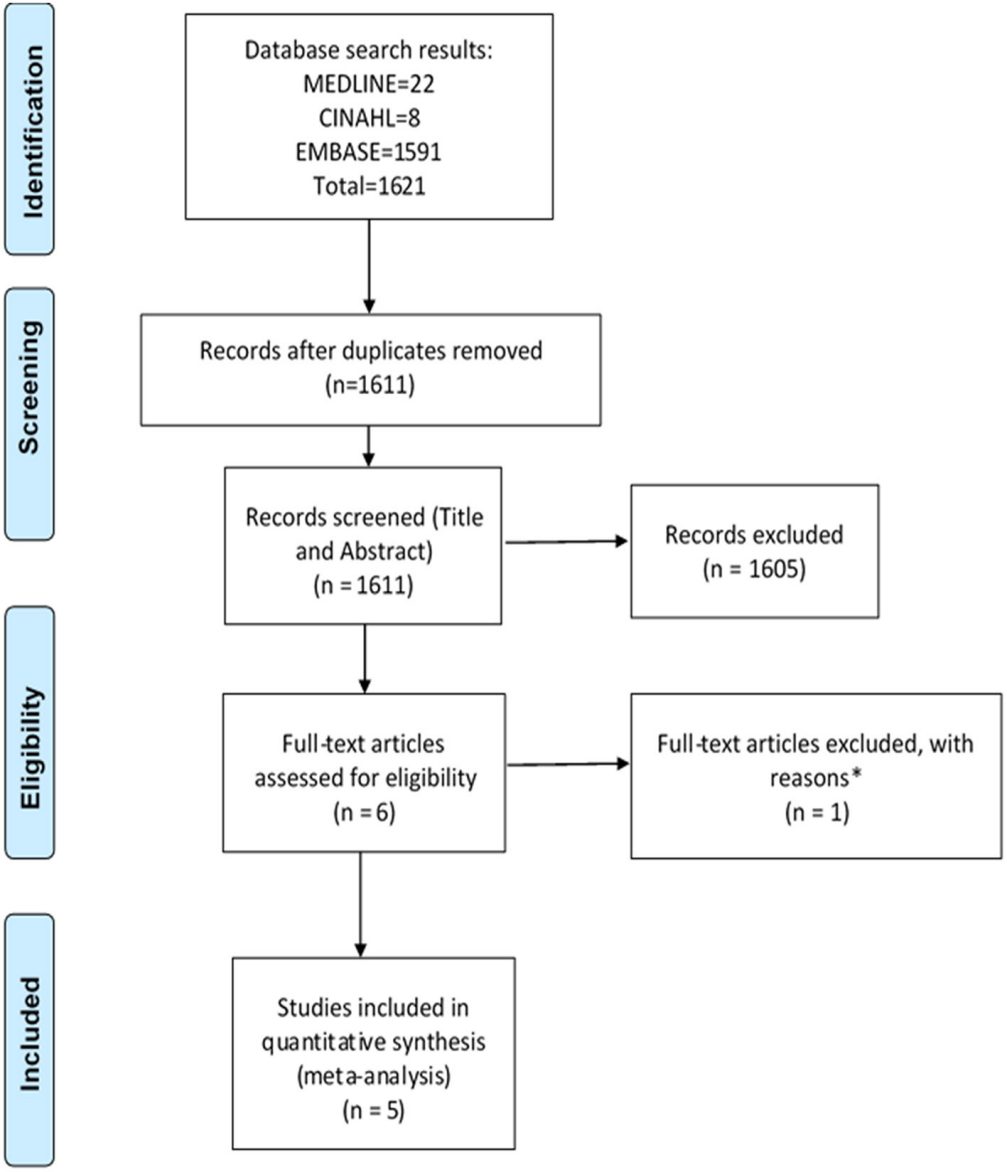
PRISMA flow diagram of literature search and included studies on hospital admissions. Adapted from Liberati et al. (2009). *Excluded studies with reasons can be found in Table 1.

**Figure 2:**
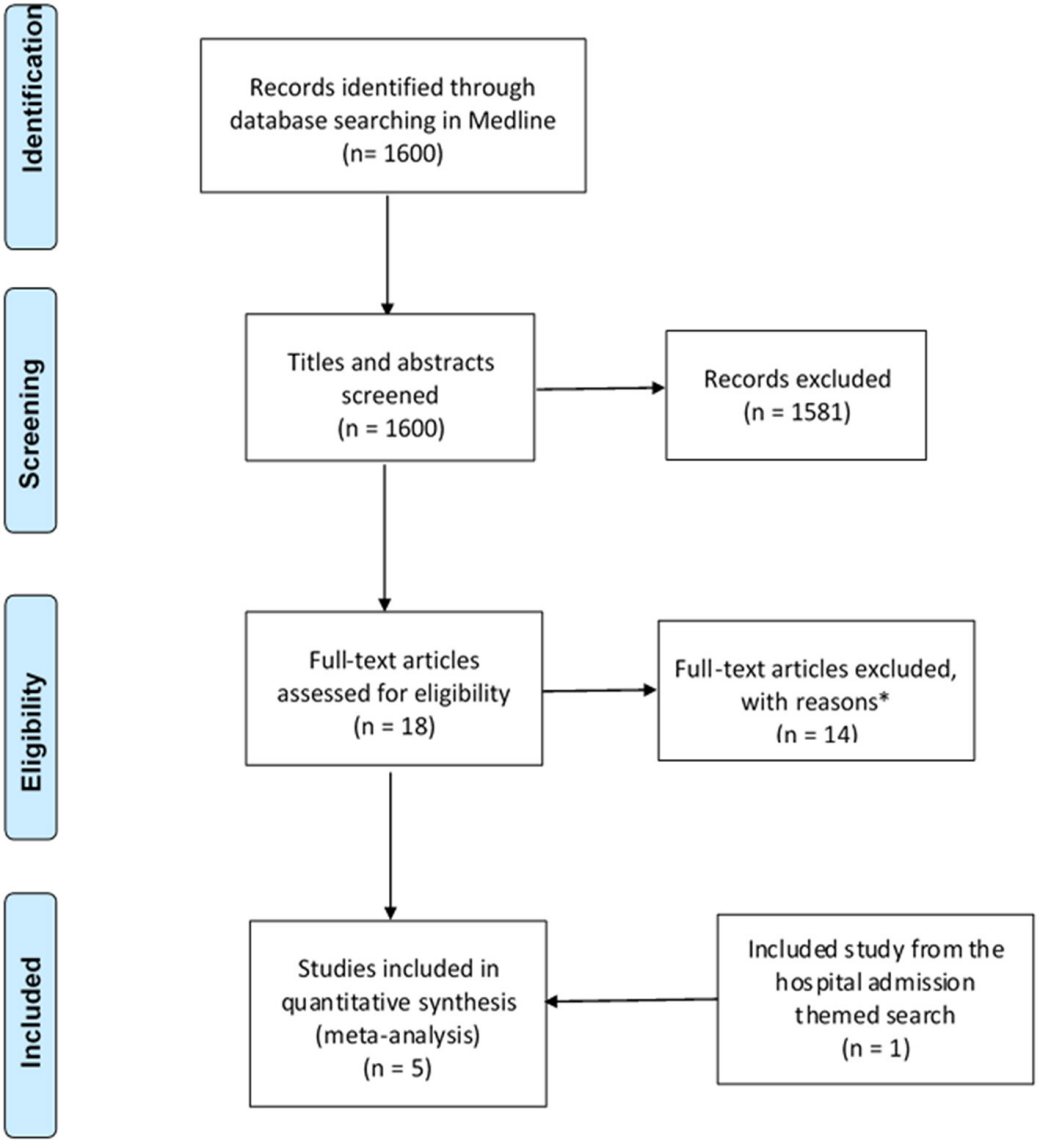
PRISMA flow diagram of literature search and included studies on medication changes themed studies. Adapted from Liberati et al. (2009). *Excluded studies with reasons can be found in Table 2.

The search strategy identified a total of 3,221 studies for both “hospital admissions” (*n* = 1,621) and “medication changes” (*n* = 1,600) themes (Figures 1, 2). Duplicates were removed and the remaining studies underwent a two-stage screening process that excluded 3,186 (at title and abstract screening stage) and 15 studies at the full-text articles stage (see Tables 1, 2 for excluded studies) in total from both themes. Five hospital admissions and five medication change themed studies were identified that met inclusion criteria that were amenable for data extraction (Tables 3, 4).

**Table 1:** Characteristics of excluded hospital admission theme studies.

**Reference**	**Reason for exclusion**
Kim et al. (2012)	PGx was not applied for the purpose of guiding pharmacotherapy.

**Table 2:** Characteristics of excluded medication changes themed studies.

**References**	**Reason for exclusion**
Carere et al. (2017)	Genetic testing to guide non-prescribed medications (i.e., over-the-counter medication or medications purchased online without a prescription) and PGx testing for the purpose of self-medication.
Ellis et al. (2019)	Non-human DNA samples tested.
Perlis et al. (2020)	Hospital admission or medication change not measured.
Povsic et al. (2019)	Modeling of impact on hospital admissions or medication changes, rather than the actual impact.
Vassy et al. (2018)	This is a study protocol
Arwood et al. (2020)	No comparison group receiving non-PGx guided therapy
Chialda et al. (2008)	No comparison group receiving non-PGx guided therapy
Collins et al. (2020)	No comparison group receiving non-PGx guided therapy
Ielmini et al. (2018)	No comparison group receiving non-PGx guided therapy
Lorizio et al. (2011)	No comparison group receiving non-PGx guided therapy
Papastergiou et al. (2017)	No comparison group receiving non-PGx guided therapy
Patel et al. (2020)	No comparison group receiving non-PGx guided therapy
Ruddy et al. (2013)	No comparison group receiving non-PGx guided therapy
Sharma et al. (2017)	No comparison group receiving non-PGx guided therapy

**Table 3:** Summary of study characteristics for medication change theme.

**Author (Year), Country**	**Study Design and number of participants**	**Mean age of participants (years old)**	**Specialty and setting**	**Single-gene or panel test**	**CDST used**	**Genes tested**	**PGx testing strategy**	Pharmacist with reviewing role*	**Outcome measure: Medication changes/clinical improvement**
Brixner et al. (2016), US	Prospective observational cohort study PGx-tested arm: 205 TAU arm: 820	PGx-guided arm: 75 TAU arm: 74	Cardiology, primary care, and internal medicine Outpatient	Panel test	YouScript®	CYP2C9, CYP2D6, CYP2C19, CYP3A4, CYP3A5, VKORC1	Polypharmacy patients currently or initiating on treatment with medication that has significant drug-gene interaction, were recruited to both arms of the study and only PGx tested in the PGx-tested arm.	Yes	The study reports that there was an average of two recommendations per patient and 46% of 381 test recommendations were followed.
Hall-Flavin et al. (2013), US	Prospective cohort study PGx-tested arm: 114 TAU arm:113	PGx-guided arm: 41 TAU arm: 44	Psychiatry Outpatient psychiatric practice	Panel test	GeneSight	CYP2D6, CYP2C19, CYP1A2, SLC6A4, HTR2A	Patients with major depressive disorder in both arms of the study were PGx tested, but only in the PGx-tested arm were the prescribers for each patient provided with the PGx test results for clinical use.	No	Medication changes occurred in 76.8% of participants from the PGx tested group and 44.1% of participants from the unguided group. 10 (55.6%) out of 18 unguided patients with significant drug-gene interactions had medication changes and 15 (93.8%) out of 16 PGx tested patients with the same interaction category had medication changes. The PGx tested group, experienced greater percent improvement in depression scores from baseline on all three depression instruments (HAMD-17, *P* < 0.0001; QIDS-C16, *P* < 0.0001; PHQ-9, *P* < 0.0001) compared with the TAU group. Eight-week response rates were higher in the PGx tested group than in the TAU group on all three measurements depression rating tools (HAMD-17, *P* = 0.03; QIDS-C16, *P* = 0.005; PHQ-9, *P* = 0.01).
Thase et al. (2019), US	RCT PGx-tested arm: 899 TAU arm:900	PGx-guided arm: 48 TAU arm: 49	Psychiatry Outpatients	Panel test	GeneSight	CYP1A2, CYP2C9, CYP2C19, CYP3A4, CYP2B6, CYP2D6, HTR2A, SLC6A4	Patients with major depressive disorder who had an inadequate response to at least one psychotropic medication were randomized to the PGx-guided arm or TAU. Patients from both arms were PGx tested but only prescribers for patients in the PGx-tested arm were provided with PGx test results for clinical use.	No	Medication changes during the first 8 weeks of treatment were significantly more common in the tested arm (65.8%; 235/357) than in the TAU arm (52.3%; 225/430) (*P* < 0.001). Among patients who switched medications, HDRS-17 scores decreased by 30.0% from baseline to week 8 in the tested arm compared to 22.3% in TAU (*p* = 0.011).
Tuteja et al. (2020), US	RCT PGx-tested arm: 252 TAU arm:257	PGx-guided arm: 63 TAU arm: 63	Cardiology Outpatients, inpatients and emergency department	Single-gene test	Decision support provided using CPIC guidelines	CYP2C19	Patients undergoing a percutaneous coronary intervention requiring antiplatelet therapy, were randomized to a PGx-guided arm or TAU. Only the patients in the PGx-tested arm were PGx tested.	No	Medication changes (switch from clopidogrel to alternative antiplatelets prasugrel/ticagrelor) occurred in 30% of participants within the PGx-guided arm and 21% of participants in the TAU arm. while 47% were started on clopidogrel.
Winner et al. (2013), US	RCT PGx-tested arm: 26 TAU arm:25	PGx-guided arm: 51 TAU arm:48	Psychiatry Outpatient clinic	Panel test	GeneSight	CYP2D6, CYP2C19, CYP1A2, SLC6A4 and HTR2A	Patients newly diagnosed with a major depressive disorder were randomized to a PGx-tested arm or TAU arm. Patients in both arms were PGx tested but only the prescribers in the PGx-tested arm were provided with the PGx test results for clinical use.	No	Medication changes occurred in 53% of the participants in the PGx-guided arm and 58% of participants in the TAU arm. Among PGx tested patients on a medication with significant gene-drug interaction (red bin category) at baseline, 100% of the medications were changed over the 10-week observation period. By comparison, 50% of the TAU patients taking red bin medications at baseline were switched or dose adjusted (*p* = 0.02). Improvement in depressive symptoms (HAMD-17) for patients that were PGx tested was higher than in the TAU patient group.

*PK, pharmacokinetics; PD, pharmacodynamics. Intervention arm is the group of participants that had PGx testing with the intention to receive PGx -guided pharmacotherapy. TAU, treatment-as-usual arm. Number of participants are the number of participants allocated to both study arms (intervention and control group). Specialty and setting are the data sources. CDST, clinical decision support tool; CPIC, Clinical Pharmacogenetics Implementation Consortium.HAMD-17/HDRS-17 is used as a tool to measure control of depressive symptoms*.

**Table 4:** Summary of study characteristics for hospital admission theme.

**Author (Year), Country**	**Study design and number of participants**	**Mean age of participants (years old)**	**Specialty and setting**	**Single-gene or panel test**	**CDST used**	**Genes tested**	**PGx test strategy**	**Pharmacist with reviewing role**	**Outcome measure: hospitalization/ED visits**
Brixner et al. (2016), US	Prospective observational cohort study PGx-tested arm: 205 TAU arm: 820	PGx-guided arm: 75 TAU arm: 74	Cardiology, Primary care, and Internal Medicine Outpatient	Panel test	YouScript® system	CYP2C9, CYP2D6, CYP2C19, CYP3A4, CYP3A5, VKORC1	Polypharmacy patients currently or initiating on treatment with medication that has significant drug-gene interaction, were recruited to both arms of the study and only PGx tested in the PGx-tested arm.	Yes	At 4 months post-enrollment. Hospitalization rate: 9.8% of patients in the tested arm vs. 16.1% in the TAU arm. Relative risk = 0.61, *p* = 0.027 ED visits were 4.4% of patients in the tested arm vs. 15.4% in the untested arm. Relative risk = 0.29, *p* = 0.0002
Elliott et al. (2017), US	RCT PGx-tested arm: 57 TAU arm: 53	PGx-guided arm: 77 TAU arm: 75	Elderly care Home health agency	Panel test	YouScript® system	CYP2C9, CYP2D6, CYP2C19, CYP3A4, CYP3A5, VKORC1	Patients initiated with at least one medication with potential for significant drug-gene interaction were recruited to the study and randomized to the PGx-tested arm or TAU arm. Only patients in the PGx tested arm were PGx tested.	Yes	At 60 days post-discharge. The mean number of re-hospitalizations per patient was 0.33 (tested) vs. 0.7 (TAU). Relative risk = 0.48, *p* = 0.007 ED visits were 0.39 (tested) vs. 0.66 (TAU). Relative risk = 0.58, *p* = 0.045 Composite number of re-hospitalization and ED visits was 0.54 (tested) vs. 1.04 (TAU)
Epstein et al. (2010), US	Prospective Observational cohort study PGx-tested arm: 896 TAU arm (historical control): 2,688	PGx-guided arm: 65 TAU arm (historical control): 65	Anticoagulation Outpatient	Panel test	Not reported	CYP2C9 and VKORC1	Patients new to warfarin treatment were PGx tested and compared to a TAU arm that were not PGx tested.	No	Overall hospitalization-Hazard ratio = 0.69, *p* = 0.001 Hospitalizations for bleeding of thromboembolism- Hazard ratio = 0.72, *p* = 0.029
Perlis et al. (2018), US	Retrospective case control design PGx-tested arm: 817 TAU arm: 2,745	PGx-guided arm: 41 TAU arm: 39	Psychiatry Outpatients	Panel test	Not reported	Ten genes with only three reported- CYP2D6, CYP2C19, CYP3A4	Patients with a mood or anxiety disorder were PGx tested in the PGx-tested arm and compared to patients with the same diagnosis in the TAU arm that were not PGx tested.	Yes- pharmacist was available for additional interpretation if required by clinician	6-month follow-up period. The mean number of inpatient hospitalizations per patient is 0.07 (tested) vs. 0.17 (TAU). 57.9% difference. *P* < 0.0001 The mean number of inpatient hospitalizations for non-mood disorders is 0.05 (in tested patients) vs. 0.14 (in TAU patients). 65.5% difference. *P* < 0.0001 The mean number of ED visits is 0.19 (in tested patients) vs. 0.33 (in TAU patients). 40.4% difference. *P* < 0.0001
Ruaño et al. (2020), US	RCT PGx-tested arm: 1,459 TAU arm: 477	Median age 40	Psychiatry Psychiatric Hospital	Single-gene testing	Clinical Evaluation Monitoring System (CEMS) and Epic EMR	CYP2D6 (21 common variants	Patients with major depressive disorder were randomized to the PGx -guided arm where patients received PGx tests or TAU where patients did not receive PGx test.	No	Readmission rate within 30 days post-discharge is 10.1% (99/982) for tested patients and 9% (43/477) for patients receiving TAU.

*PK, pharmacokinetics; PD, pharmacodynamics. Intervention arm is the group of participants that had PGx testing with the intention to receive PGx -guided drug therapy. TAU, treatment-as-usual arm. Number of participants are the number of participants allocated to both study arms (intervention and control group). Specialty and setting are the data sources. CDST, clinical decision support tool*.

### Quality Assessment

The included studies were assessed for risk of bias (methodological quality) using the CASP checklists for three types of study designs: randomized controlled trials, case-control study and cohort studies. The bias risk assessment results of the studies show that the overall quality of the studies is good. The main problems are the identification of all-important confounders in the cohort studies and blinding in the RCTs as shown in Supplementary Tables 1, 2. Confounding factors, such as kidney and liver function, are not considered in the cohort studies, although they are potential risk factors for adverse drug reactions (Brixner et al., 2016). One RCT was a single-blinded study, two open-label studies (Elliott et al., 2017; Perlis et al., 2018), one triple blinded study (Hall-Flavin et al., 2013) and one double-blinded study (Tuteja et al., 2020).

### Summary of the Study Characteristics

The included studies were all published between 2010 and 2020, all conducted in the USA. A summary of study characteristics for medication change themed studies and hospital admission themed studies are shown in Tables 3, 4, respectively. Five studies reported hospital admission, four studies reported medication changes and one study (Brixner et al., 2016) reported both hospital admissions and medication changes.

The reviewed studies included both observational and experimental research methodologies: one case-control study (Perlis et al., 2018), three cohort studies and five randomized controlled studies.

The average age of the participants in all the studies ranged from 40 to 75 years old (Tables 3, 4). The studies covered psychiatry and cardiovascular related conditions for 55% (*n* = 5) and 33% (*n* = 3) of the studies, respectively, while 22% (*n* = 2) of the studies were cross-speciality, consisting of a combination of internal medicine, primary care or elderly care. The type of testing most used were panel tests (77% of all studies reviewed, 7/9 that test for multiple genes and their variants rather than single-gene tests). Both pharmacokinetic and pharmacodynamic genes were tested for 77% (*n* = 7) of all studies reviewed. Of all the included studies, pharmacists reviewed PGx test results in only two studies (Brixner et al., 2016; Elliott et al., 2017) and consultation with a pharmacist was reported as optional in one study (Perlis et al., 2018). PGx testing in the included studies occurred as part of the study design so that hospitalization and medication changes in the PGx tested arm of the study can be compared to the TAU arm. PGx testing in the included studies were reactive and included in the study designs. For the medication change themed studies, two types of reactive PGx testing were observed: 1) PGx testing occurring post-diagnosis with an indicated treatment 2) PGx testing occurring in response to poor efficacy or poor tolerability to medication(s). Three out of the five studies selected a population with a specific diagnosis or undergoing a procedure that required subsequent pharmacotherapy (Hall-Flavin et al., 2013; Winner et al., 2013; Tuteja et al., 2020). The remaining two studies, Brixner et al. (2016) and Thase et al. (2019), selected patients with a history of poor drug response to psychotropic medication and polypharmacy patients taking medication with potential for significant drug-gene interactions, respectively.

### Generalizable Steps in PGx Process

As described above the nine studies were diverse in nature. Five studies dealt with hospital admission four studies dealt with medication changes (Hall-Flavin et al., 2013; Winner et al., 2013; Thase et al., 2019; Tuteja et al., 2020) and one study dealt with both hospital admissions and medication changes (Brixner et al., 2016).

**Figure 3:**
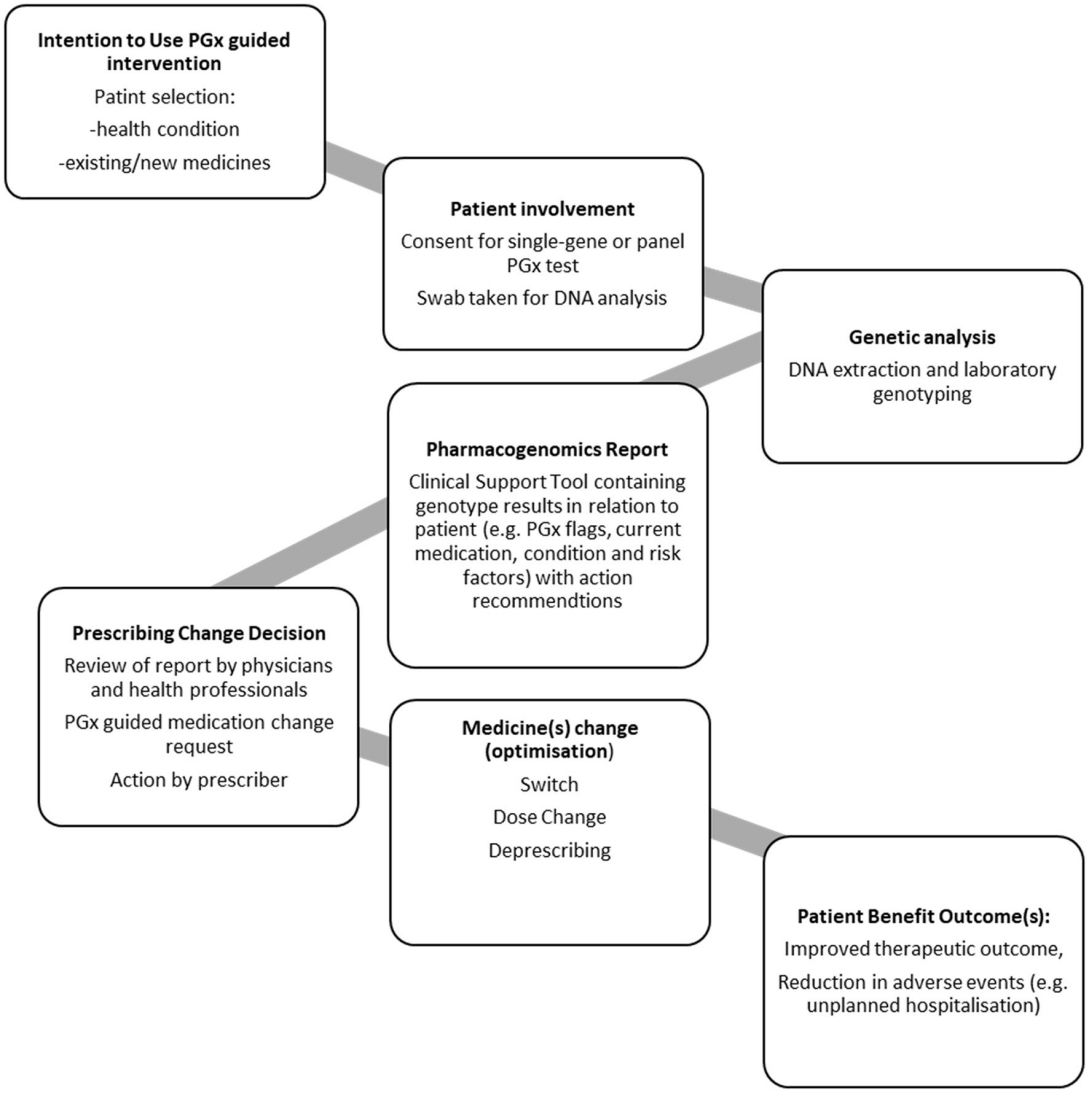
PGx General process model for pharmacogenomic testing as an intervention.

To aid derivation of themes for analysis, first a process diagram was produced; derived from the steps described in each of the studies (Figure 3). This PGx general process model outlines the steps required for a PGx intervention that can prompt medication changes, patient benefit and reduce hospital admission.

### Analysis of Included Studies in Relation to a PGx General Process Model

#### Patient Groups

Patients included in studies presented with a range of health conditions, but the focus was largely on those who require polypharmacy regimens and long-term medication such as warfarin, clopidogrel, antidepressants and antipsychotics (Epstein et al., 2010; Hall-Flavin et al., 2013; Winner et al., 2013; Brixner et al., 2016; Elliott et al., 2017; Perlis et al., 2018; Thase et al., 2019; Ruaño et al., 2020; Tuteja et al., 2020). The most common speciality studied was psychiatry, accounting for 55% (5/9) of the included studies. The remaining studies covered cardiology, primary care, elderly care, anticoagulation, and internal medicine.

The type of PGx associated medications reported as used by participants in all studies suggests patients in all the studies have chronic conditions that require the use of medication long-term. Two of the hospitalization themed studies, Brixner et al. (2016) and Elliott et al. (2017) specifically recruited polypharmacy patients, defined in the study as patients taking ≥3 medications or participants taking an average of 11 medications, respectively. Perlis et al. (2018) and Ruaño et al. (2020) recruited patients that require psychotropic medication for major depressive disorder and mood/anxiety disorders, respectively. From the medication change themed studies, Tuteja et al. (2020) focused on patients that require antiplatelet therapy with clopidogrel or its alternative ticagrelor/prasugrel, after undergoing percutaneous coronary intervention (PCI) with stent implantation. Hall-Flavin et al. (2013), Winner et al. (2013) and Thase et al. (2019) focused on patients that require treatment with psychotropic medication for major depressive disorder.

#### Involvement of the Patient and Consent

While the consent requirements for routine PGx have not yet been established, the patients in all the studies provided informed consent on entry to the included studies (Epstein et al., 2010; Hall-Flavin et al., 2013; Winner et al., 2013; Brixner et al., 2016; Elliott et al., 2017; Perlis et al., 2018; Thase et al., 2019; Ruaño et al., 2020; Tuteja et al., 2020).

#### DNA Sampling and Genetic Analysis

In each study, patients provided a buccal swab. In some cases, the swabs were sent to an external laboratory for DNA isolation and gene analysis, whereas in some studies, this was conducted in-house.

There is not currently a single standardized test or panel for PGx, so each of the studies reported on different genotypes that were selected to be appropriate to their patient population. In the included studies, both single gene and panel PGx tests were employed. The genotypes tested, and where published, are included in Tables 3, 4, with one study (Perlis et al., 2018), reporting on 3 of the 10 genes included in their analysis panel.

#### Laboratory Reporting of Genotype and Laboratory/Provider Recommendations

Currently, there is no standardization of how genotype is reported and how that information is associated with a patient's record so this will vary according to clinical setting and health economy. In the hospitalization themed studies, clinical decision support tools were utilized to report genotype results and provide prescribing recommendations in Brixner et al. (2016), Elliott et al. (2017), Perlis et al. (2018), and Ruaño et al. (2020). In Epstein et al. (2010), no details were provided on how genotype results and prescribing recommendations were provided to the clinicians involved in patient care. In the medication change themed studies, Hall-Flavin et al. (2013), Winner et al. (2013) and Thase et al. (2019) all used a clinical decision support tool to report genotype results and provide recommendations. Tuteja et al. (2020) did not report the use of a support tool but prescribers were provided with prescribing decision support, using a widely used PGx prescribing guideline known as CPIC (Clinical Pharmacogenetics Implementation Consortium).

#### Prescribing Recommendations

PGx reports aim to provide information for clinical teams and prescribers to support medicine optimization such as medications switch, change of dose or deprescribing. These reports require review by team members with appropriate expertise to understand and implement the recommendations whilst exercising clinical judgement. In all the studies, prescribing based on PGx guidance was at the discretion of the prescriber (Epstein et al., 2010; Hall-Flavin et al., 2013; Winner et al., 2013; Brixner et al., 2016; Elliott et al., 2017; Perlis et al., 2018; Thase et al., 2019; Ruaño et al., 2020; Tuteja et al., 2020). Not all the studies described the process by which PGx reports were reviewed once received in the clinic (Epstein et al., 2010; Hall-Flavin et al., 2013; Winner et al., 2013; Thase et al., 2019; Ruaño et al., 2020; Tuteja et al., 2020). In some studies, a pharmacist reviewed the report (Brixner et al., 2016; Elliott et al., 2017) or was available for consultation (Perlis et al., 2018). None of the studies reported patient's involvement in decision-making.

The medication change themed studies suggest that medication changes occurred as a result of the provision of PGx information (Hall-Flavin et al., 2013; Winner et al., 2013; Brixner et al., 2016; Thase et al., 2019; Tuteja et al., 2020). However, only one of the published studies provide detail on the proportion of PGx recommendations that were implemented: Brixner et al. (2016) reported that 46% of 381 test recommendations were followed but did not report on the number of patients that had medication changes.

#### Medication Changes

Four of the five medication themed studies provided data on the number of patients with changes to medications that occurred in the PGx-guided arm during the study period, compared to the TAU arm. We note, as above, that these studies were varied in relation to their settings, the number of genes tested, and the number of medications that were likely to be able to be changed as a result of PGx guidance. The analysis included all measures of the number of patients that received medication changes as derived from the publications, with per protocol analysis undertaken for Hall-Flavin et al. (2013) and Thase et al. (2019) so to exclude from the analysis participants who withdrew.

**Figure 4:**
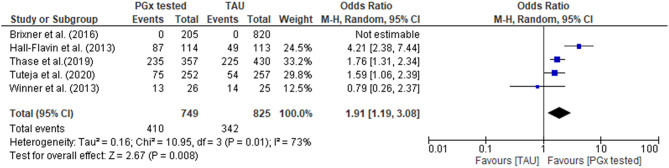
Forest plot showing the effect of PGx testing on medication changes compared to treatment-as-usual (TAU). The number of “events” are the number of participants that received a medication change out of the total number of participants. Brixner et al. (2016) did not report the number of participants that received medication changes in both the PGx tested and the TAU arm, was not included in the analysis.

In the PGx tested groups, 54.7% of patients had a medication change, compared to 41.5% of changes in the TAU group, representing a 32% increase in medication changes overall. The quantitative data were combined for meta-analysis (Figure 4) showing a statistically significant increase in medications changes in the PGx arm across 749 patients in the PGx-tested arm and 825 patients in the TAU arm, with an odds ratio of 1.91 (*Z* = 2.67, *p* = 0.008). However, given the small number of studies included and the diversity of settings, the heterogeneity as measured by *I*^2^ was high (73%). The influence analysis using the Baujat plot (Supplementary Figure 1A) highlighted that the Hall-Flavin et al. (2013) study contributed much to the overall heterogeneity whilst at the same time was not very influential on the overall combined effect. Furthermore, in Supplementary Figure 1B we show the Viechtbauer and Cheung influence analyses (Viechtbauer and Cheung, 2010) which again shows that Hall-Flavin et al. (2013) has a large influence on heterogeneity as it is has very extreme values in these plots as highlighted by the red spot.

#### Hospital Admissions

Five studies measured hospital admission, from a variety of causes, as an endpoint of PGx-guided intervention. We note, as above, that these studies were varied in relation to their settings, the number of genes tested, and the medications changed as a result of PGx guidance.

**Figure 5:**
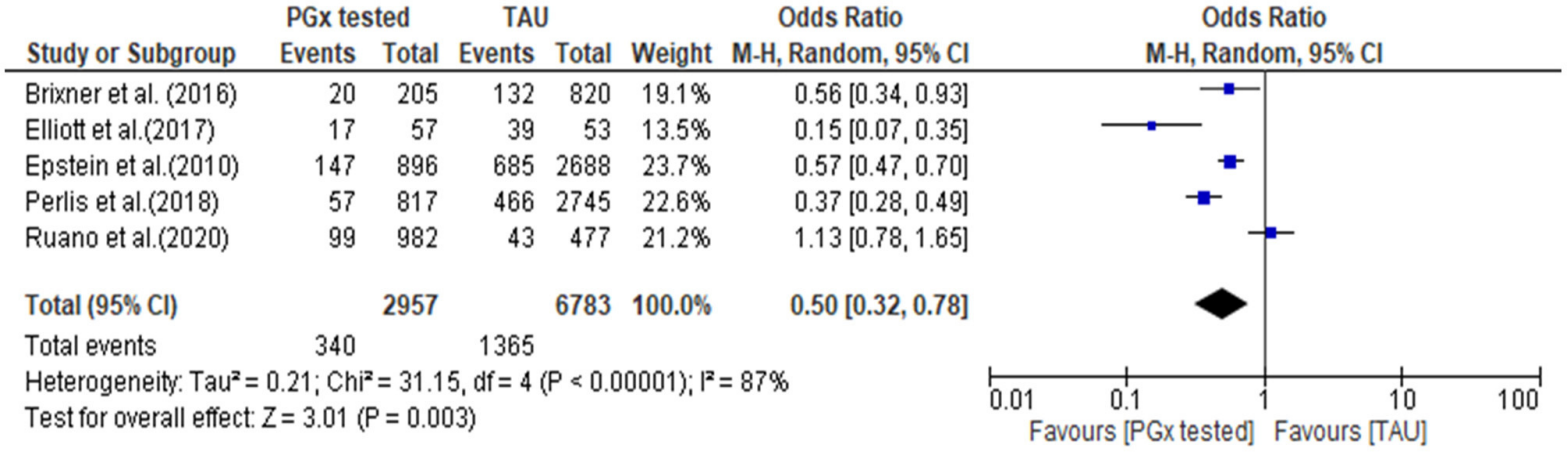
Forest plot showing the effect of PGx testing on hospitalization compared to treatment-as-usual. 95% CI, 95% confidence interval. The number of “events” are the number of participants who were hospitalized during the reporting period of the study.

The quantitative data from each individual study for all-cause hospitalization were available for meta-analysis, as presented in a forest plot and associated table (Figure 5). The results of the hospital admission themed analysis show that a reduction in unplanned hospital admission occurred in four out of the five studies. These data show that all-cause hospital admissions occurred significantly less frequently in the PGx tested arm incorporating data from 2,957 patients in the PGx-tested arm and 6,783 patients in the treatment as usual arm being significant, with an odds ratio of 0.5 (*Z* = 3.01 *p* = 0.003). In the PGx tested groups, 11.5% of patients had a hospital admission, compared to 20.1% of changes in the TAU group, representing a 43% reduction in hospital admissions for people receiving PGx testing in these trials. However, given the small number of studies included and the diversity of settings, the heterogeneity as measured by *I*^2^ was high (87%). The influence analysis using the Baujat plot (Supplementary Figure 2A) highlighted that the Ruaño et al. (2020) study contributed much to the overall heterogeneity and was very influential on the overall combined effect. Furthermore, in Supplementary Figure 2B we show the Viechtbauer and Cheung influence analyses (Viechtbauer and Cheung, 2010) which again show that Ruaño et al. (2020) has a large influence on heterogeneity as it has extreme values in these plots (highlighted by the red spot).

Further details of the effect of PGx testing on all-cause hospitalization measured in the studies are presented in Table 5 together with data on unplanned hospitalization and outpatient visits where that was measured and reported in the studies. For the three studies which reported unplanned hospital admissions, there were consistent reductions in unplanned hospitalization. Two studies measured outpatient visits (Brixner et al., 2016; Perlis et al., 2018), with one study reporting a large increase in outpatient visits among people receiving a PGx test (Brixner et al., 2016) and one study reporting a slight decrease (Perlis et al., 2018).

**Table 5:** Risk of hospitalization in PGx-tested arm and control (treatment-as-usual) arm.

**Author (Year), Country**	**All-cause hospitalization (%)**	**Emergency department visit (%)**	**Outpatient visit (%)**
Brixner et al. (2016), US	Risk was reduced by 39% relative to the control group. (RR = 0.61) 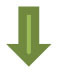	Risk was reduced by 71% relative to the control group. (RR = 0.29) 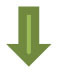	Outpatient visits increased by 97% relative to the control group. (RR = 1.97) 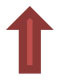
Elliott et al. (2017), US	Risk was reduced by 52% relative to the control group. (RR = 0.48) 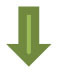	Risk was reduced by 42% relative to the control group. (RR = 0.58) 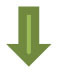	Not measured
Epstein et al. (2010), US	31% less likely to be hospitalized than patients in the control group. (HR = 0.69) 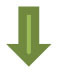	Not measured	Not measured
Perlis et al. (2018), US	58% difference between the intervention and control group. (RR = 0.42) 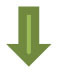	40% difference between the intervention and control group. 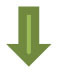	13% difference between the intervention and control group. 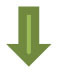
Ruaño et al. (2020), US	No significant difference between the intervention and control group. 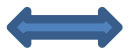	Not measured	Not measured

*The study outcomes are presented as relative risk, hazard ratio and percentage difference between the intervention and control group. Reduction in hospital admission is denoted by the green downwards pointing arrow (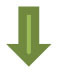), increase in outpatient visits denoted by the red upward pointing arrow (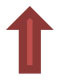) and no signicant difference is denoted by the blue double bidirectional arrow (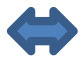)*.

## Discussion

This study shows that currently, there are a limited number of studies that compared PGx-guided pharmacotherapy with treatment as usual in relation to the measurable patient outcomes of medication changes and hospitalization. The published evidence demonstrates the potential of PGx testing in a range of settings to produce patient benefit. This is demonstrated both from the narrative synthesis and also the meta-analysis achieved through combining the results of all studies with similar outcome measures. The analysis shows that PGx testing can produce substantial benefits in patient populations, by substantially increasing the number of medication changes that could potentially result in a reduction in serious adverse events as measured by hospital admission. Hospital admissions were halved across the interventions included in this study. These findings contribute to evidence for the clinical utility of PGx testing which is often lacking in literature and altogether provide strong proof-of-concept for the clinical utility of PGx testing in guiding medication changes that result in clinical improvements and that PGx testing can potentially reduce hospitalization.

Medication changes occuring as a result of undesirable drug response, are to be expected in both PGx tested and TAU arms of the study, and we showed (Figure 4) that PGx resulted in more medication changes. In this study we have treated medication changes as a desirable outcome indicating that prescribers have acted on PGx information to review medications already in use by patients to avoid undesirable or suboptimal drug resposnes. In the studies analyzed, PGx tests were conducted on patients who were already taking medications to treat diagnosed disease. Therefore, in the studies reviewed here, PGx information should prompt medication optimisation and change. This is borne out in studies which reported on this: where PGx testing was done it revealed numerous patients taking medcations with predicted significant gene-drug interactions who were at risk of poor drug response (Hall-Flavin et al., 2013; Winner et al., 2013; Thase et al., 2019). Therefore, a switch or dose adjustment in these cases is a positive outcome. It is important to note, however once PGx testing becomes a routine part of prospective prescribing decision making, it would be expected that fewer medication changes would occur in patients receiving PGx-guided treatment, as patients should be more likely to receive medications and doses of those medications that are effective and have fewer side effects than current treatment as usual. A pre-emptive approach did not take place in these studies but is being developed for storage of PGx information and its future use (Relling and Evans, 2015; Lazaridis, 2019). Current implementation studies with this intention are underway, for example a 5-year prospective study started in 2016 known as PREPARE (PREmptive Pharmacogenomic testing for preventing Adverse drug REactions [ADRs]), conducted by the Ubiquitous Pharmacogenomics (U-PGx) consortium (Manson et al., 2017). The study aims to generate evidence, using multi-center RCTs, for the effect of pre-emptive PGx testing on reducing ADRs by considering multiple drugs, genes, ethnicities and health care systems across seven European countries (Manson et al., 2017).

We note that it would be premature to draw a definitive conclusion on the impact of PGx testing on medication changes and hospital admissions, due to the nature of the studies that are currently available. There are only a small number of published studies which have included medication changes and hospital admission end points. Whilst the published studies all are well-designed and have reported appropriatetly, the studies all have different designs. This leaves some significant gaps in our understanding of how to ensure effectiveness of a PGx intervention. Aspects that require consideration are mapped in the generalized process model (Figure 3), namely the patients who would most benefit from PGx testing, the genetic variants to be routinely included in tests, how the information is provided to clinical teams, and whether the recommendations are taken up.

One key question is whether interventions can be used with larger populations with a wider range of conditions, for example older patients on multiple medications. Improvement in clinical outcome consists of symptom improvement and higher response and remission rates. The extent of improvement possible from PGx testing is dependent on the existing medications a patient is on, their own genotype and the number of significant PGx variants they have which predict a sub-optimal or harmful response to a medication. Therefore, the choice of patients for PGx-guided intervention is important. The majority of the included studies focussed on a specific condition or group of patients, and chose a small number of genes to test. We note that clinical outcome improvements were observed particularly in those who were taking medications with significant drug-gene-interactions at baseline and then switched to medications with no drug-gene interactions. Thase et al. (2019) found that 66.4% of PGx-tested patients switched from medications with significant drug-gene interactions to medications with no drug-gene interactions and saw a 7.6% improvement in depressive symptoms (HDRS-17 scores) at 8 weeks from baseline. Patients taking medications with significant or modest drug-gene interactions at baseline, reported greater clinical improvement in the PGx-tested arm than TAU arm in Winner et al. (2013) and Hall-Flavin et al. (2013). Thus, studies which are less specifically targeted to patients who are taking medications with significant drug-gene interactions may well have a smaller impact on medication change compared to the studies that were included and analyzed here. However, it is reassuring that the studies of Brixner et al. (2016) and Elliott et al. (2017) which studied older patient populations in non-specialist settings both provided positive evidence that PGx testing reduces hospitalization. Furthermore, a reduction in hospitalization amongst PGx tested patients, infers the acceptance of PGx-guided recommendations by prescribers. Hall-Flavin et al. (2013) reported that PGx tested patients identified to be taking medications categorized with significant drug-gene interactions, were more likely to experience changes to their medications than non-PGx tested patients taking medications in the same category. PGx testing can help healthcare professionals predict serious drug therapy problems for individual patients and thus prescribers are more likely to accept subsequent PGx-guided recommendations.

The majority of the included studies, except for Ruaño et al. (2020) and Tuteja et al. (2020), recruited outpatients and inform a care pathway with patients receiving PGx testing in a primary care setting. We note that medication changes and new medication initiations often occur in hospital, but PGx testing for inpatients in secondary care is not yet well-represented in the literature. Therefore, more studies are required to investigate how PGx testing could be used for hospital inpatients and how a care pathway that incorporates PGx testing would work in practice in these settings.

Improved medications optimisation and reduction in adverse effects, particularly hospitalizations as well as benefiting patients, offer potential cost-savings for health systems which are beyond the scope of this review. However, we note that one study reported a 39 and 71% reduction in the risk of inpatient hospitalization and emergency department visit, respectively, but a 97% increase in outpatient visits (Brixner et al., 2016), most likely due to multiple appointments required to make changes to the patient's medications. Thus, the impact on other parts of the health systems should be a consideration for cost-effectiveness studies and the deisgn of a care pathway that considers both the current workload of healthcare professionals and patient convenience and benefit.

Genotyping was the genomic testing technology applied in the included studies and the technology focuses on predetermined genetic variants. This technology utilizes biochemical assays and is usually cheaper than next generation sequencing in sequencing variants in the tens to low hundreds (Mitropoulou et al., 2020). For PGx-guided pharmacotherapy to benefit patients, PGx tests should be predictive for the clinically important drug-gene interactions relevant to the patient and should contribute to improved health outcomes. For that reason, the PGx tests selected should cover all genetic variants with significant evidence. In these proof of concept studies, this was not always the case: for example Ruaño et al. (2020) in their study focused on psychiatry patients and tested a single gene, CYP2D6 whilst there are currently other genes, including CYP2C19 with significant evidence for variants that affect response to medications in psychiatry (Hall-Flavin et al., 2013; Perlis et al., 2018; Thase et al., 2019). Testing for just one gene in Ruaño et al. (2020) could therefore underestimate the effect that PGx testing could have on reducing hospitalization in patient populations. This seems likely, since testing for genes CYP2C19 and CYP2D6 amongst other genes, Perlis et al. (2018) reports a greater reduction in hospitalization. Epstein et al. (2010) tested for common genes associated with warfarin response, namely CYP2C9 and VKORC1, before the gene CYP4F2 was recently added to CPIC guidelines and inclusion of that gene may have further improved the decrease in hospitalization observed. Conversely, in some of the studies, gene variants have been included which do not yet have consensus guidance on medications used in the condition, so their predicted effect on patient outcomes is not clear. For example the non- CYP2D6 and non-CYP2C19 genetic variants tested in Hall-Flavin et al. (2013); Winner et al. (2013); Perlis et al. (2018), and Thase et al. (2019) are not known to have an important role in psychiatry. In the future, a PGx test that includes the most clinically relevant drug-gene interactions and preferably the widest range of commonly prescribed medications, could be most useful for routine care of general patient populations. PGx panel testing for multiple medications is particularly important when considering older patients, who are more at risk of adverse drug reactions and are more likely to be prescribed multiple medications. PGx testing is particularly attractive when considering the risks associated with polypharmacy. A reactive testing approach conducted on sub-populations at most risk of adverse drug reactions, such as older people, will allow for a more targeted approach in the use of healthcare systems' finite resources.

The way in which laboratory tests are incorporated into clinical decision support tools is an essential area for research and development to ensure that the potential benefits of PGx are maximized. In this analysis, six of the included studies used proprietary software or algorithms to produce reports, and we have not found published evidence of how the predictions are constructed, their effectiveness and precision or their usefulness to prescribers and healthcare staff. Decision support tools with algorithms that take account of clinical parameters that affect response to medications (i.e., age, body-mass-index ratio, sex, renal, liver function, current medication in use) will be appropriate in delivering personalized care for patients (Turner et al., 2020). Since routine PGx-guided care is at an early stage of development, the information needs of prescribers and healthcare staff as well as their perception of PGx-guided care, need to be considered. A Greek study reported that 50% of pharmacists and medical doctors felt that they were unable to provide sufficient information to patients explaining PGx test results (Mai et al., 2014). Taking account of information needs and perception amongst this group will ensure that decision support tools can better support workflow for PGx prescribing.

Finally, we note that included studies provided little detail on whether the recommendations in the PGx report were actioned, with the exception of Brixner et al. who recorded that 46% of test recommendations were followed. This detail is essential for future PGx studies, to understand whether the medication changes were genuniely PGx-guided or changed for other reasons such as inclusion in a trial that might trigger a more complete patient medications review than “treatment as usual.” Detail of medication changes is lacking in the included studies and exploration of whether prescribers felt more comfortable in changing dose than changing medication or changing particular types of medication is also absent. This knowledge is essential to inform training for prescribers and to effectively deliver PGx-guide care. When designing implementation strategies of PGx acrosss health systems, such as the NHS, understanding how the information is constucted so that it can be understood and acted on by health professionals to implement PGx-guided medication adjustments is critical. In addition, the patient's involvement in prescribing decisions is important; patient's perspectives on care affects adherence to pharmacotherapy and how they take their medications (Joosten et al., 2008; Silva, 2012). Therefore, investigating patient's attitude to PGx testing can contribute postively in making PGx-guided care more patient-centered.

## Conclusion

Results from this study provide evidence that PGx testing increases medication changes and reduces hospitalization and also provide a proof-of-concept for the clinical utility of PGx-guided care that informs future pharmacotherapy. The small number of studies reviewed demonstrates the novelty of the review question, and our analysis contributes to identifying gaps where further research is required, including study into the perspectives of patients and prescribers to aid the design of PGx-guided care that is patient-centered.

## Data Availability Statement

The original contributions presented in the study are included in the article/Supplementary Material, further inquiries can be directed to the corresponding author/s.

## Author Contributions

VD contributed to the study conception, implementation of the research, the analysis of the results, and the writing of the manuscript. BF contributed to the planning of the narrative synthesis and the draft revisions of the manuscript. EB contributed to the writing of the manuscript. HS contributed to the study conception and draft revisions of the manuscript. GS contributed to the study conception, analysis of the results, and the draft revisions of the manuscript. MR supervised the process, contributed to the study conception, analysis of the results, and was in charge of overall direction and planning. All authors contributed to the article and approved the submitted version.

## Author Disclaimer

The views expressed in this article are those of the authors and not necessarily those of the NHS, the NIHR, or the Department of Health and Social Care.

## Conflict of Interest

The authors declare that the research was conducted in the absence of any commercial or financial relationships that could be construed as a potential conflict of interest.

## Publisher's Note

All claims expressed in this article are solely those of the authors and do not necessarily represent those of their affiliated organizations, or those of the publisher, the editors and the reviewers. Any product that may be evaluated in this article, or claim that may be made by its manufacturer, is not guaranteed or endorsed by the publisher.

## Footnotes

^1^Endnote online 2021. Clarivate.

^2^Critical Appraisal Skills Programme, CASP (Randomized Controlled Trial) Checklist. 2020.

^3^Critical Appraisal Skills Programme, CASP (Cohort Study) Checklist. 2018.

^4^Critical Appraisal Skills Programme, CASP (Case Control Study) Checklist. 2018.

^5^Review Manager (RevMan) [Computer program]. Version 5.4, The Cochrane Collaboration 2020.

## References

[B1] Alexopoulou A. Dourakis S. P. Mantzoukis D. Pitsariotis T. Kandyli A. Deutsch M.. (2008). Adverse drug reactions as a cause of hospital admissions: a 6-month experience in a single center in Greece. Eur. J. Intern. Med. 19, 505–510. 10.1016/j.ejim.2007.06.030 19013378

[B2] Alshabeeb M. A. Deneer V. H. M. Khan A. Asselbergs F. W. (2019). Use of pharmacogenetic drugs by the Dutch population. Front. Genet. 10:567. 10.3389/fgene.2019.00567 31312209 PMC6614185

[B3] Arwood M. J. Dietrich E. A. Duong B. Q. Smith D. M. Cook K. Elchynski A.. (2020). Design and early implementation successes and challenges of a pharmacogenetics consult clinic. J. Clin. Med. 9:2274. 10.3390/jcm9072274 32708920 PMC7408871

[B4] Baujat B. Mahé C. Pignon J. P. Hill C. (2002). A graphical method for exploring heterogeneity in meta-analyses: application to a meta-analysis of 65 trials. Stat. Med. 21, 2641–2652. 10.1002/sim.1221 12228882

[B5] Brixner D. Biltaji E. Bress A. Unni S. Ye X. Mamiya T.. (2016). The effect of pharmacogenetic profiling with a clinical decision support tool on healthcare resource utilization and estimated costs in the elderly exposed to polypharmacy. J. Med. Econ. 19, 213–228. 10.3111/13696998.2015.1110160 26478982

[B6] Brown S. A. Pereira N. (2018). Pharmacogenomic impact of CYP2C19 variation on clopidogrel therapy in precision cardiovascular medicine. J. Pers. Med. 8:8. 10.3390/jpm8010008 29385765 PMC5872082

[B7] Bush W. S. Crosslin D. R. Owusu-Obeng A. Wallace J. Almoguera B. Basford M. A.. (2016). Genetic variation among 82 pharmacogenes: The PGRNseq data from the eMERGE network. Clin. Pharmacol. Ther. 100, 160–169. 10.1002/cpt.350 26857349 PMC5010878

[B8] Carere D. A. VanderWeele T. J. Vassy J. L. van der Wouden C. H. Roberts J. S. Kraft P.. (2017). Prescription medication changes following direct-to-consumer personal genomic testing: findings from the Impact of Personal Genomics (PGen) Study. Genet. Med. 19, 537–545. 10.1038/gim.2016.141 27657683 PMC5362351

[B9] Chialda L. Griffith L. S. Heinig A. Pahl A. (2008). Prospective use of CYP pharmacogenetics and medication analysis to facilitate improved therapy – a pilot study. Pers. Med. 5, 37–45. 10.2217/17410541.5.1.37 29783392

[B10] Collins A. R. Kung S. Ho J. T. Wright J. A. Dammen K. C. Johnson E. K.. (2020). Pharmacogenetic testing in psychiatric inpatients with polypharmacy is associated with decreased medication side effects but not via medication changes. J. Psychiatr. Res. 126, 105–111. 10.1016/j.jpsychires.2020.05.002 32442780 PMC9441021

[B11] Deeks J. J. Higgins J. P. T. Altman D. G. (2021). Chapter 10: Analysing data and undertaking meta-analyses, in Cochrane Handbook for Systematic Reviews of Interventions, Version 6.2, eds Higgins J. P. T. Thomas J. Chandler J. Cumpston M. Li T. Page M. J. Welch V. A. (Lancaster, PA: Cochrane; University of Lancaster). Available online at: www.training.cochrane.org/handbook

[B12] Elliott L. S. Henderson J. C. Neradilek M. B. Moyer N. A. Ashcraft K. C. Thirumaran R. K. (2017). Clinical impact of pharmacogenetic profiling with a clinical decision support tool in polypharmacy home health patients: A prospective pilot randomized controlled trial. PLoS ONE 12:e0170905. 10.1371/journal.pone.0170905 28151991 PMC5289536

[B13] Ellis K. E. Nawas G. T. Chan C. York L. Fisher J. Connick E.. (2019). Clinical outcomes following the use of archived proviral HIV-1 DNA genotype to guide antiretroviral therapy adjustment. Open Forum Infect. Dis. 7:ofz533. 10.1093/ofid/ofz533 31915714 PMC6942490

[B14] Epstein R. S. Moyer T. P. Aubert R. E. Kane D. J. Xia F. (2010). Warfarin genotyping reduces hospitalization rates results from the MM-WES (Medco-Mayo Warfarin Effectiveness study). J. Am. Coll. Cardiol. 55, 2804–2812. 10.1016/j.jacc.2010.03.009 20381283

[B15] Grant M. J. Booth A. (2009). A typology of reviews: an analysis of 14 review types and associated methodologies. Health Info. Libr. J. 26, 91–108. 10.1111/j.1471-1842.2009.00848.x 19490148

[B16] Hall-Flavin D. K. Winner J. G. Allen J. D. Carhart J. M. Proctor B. Snyder K. A.. (2013). Utility of integrated pharmacogenomic testing to support the treatment of major depressive disorder in a psychiatric outpatient setting. Pharmacogenet. Genomics. 23, 535–548. 10.1097/FPC.0b013e3283649b9a 24018772

[B17] Hanlon P. Nicholl B. I. Jani B. D. McQueenie R. Lee D. Gallacher K. I.. (2018). Examining patterns of multimorbidity, polypharmacy and risk of adverse drug reactions in chronic obstructive pulmonary disease: a cross-sectional UK Biobank study. BMJ Open 8:e018404. 10.1136/bmjopen-2017-018404 29332840 PMC5781016

[B18] Harrer M. Cuijpers P. Furukawa T. Ebert D. (2021). Doing Meta-Analysis With R: A Hands-On Guide. Boca Raton, FL: Chapmann & Hall/CRC Press.

[B19] Ielmini M. Poloni N. Caselli I. Espadaler J. Tuson M. Grecchi A.. (2018). The utility of pharmacogenetic testing to support the treatment of bipolar disorder. Pharmacogenomics Pers. Med. 11, 35–42. 10.2147/PGPM.S160967 29588611 PMC5860421

[B20] Ingelman-Sundberg M. (2001). Pharmacogenetics: an opportunity for a safer and more efficient pharmacotherapy. J. Intern. Med. 250, 186–200. 10.1046/j.1365-2796.2001.00879.x 11555122

[B21] Ji Y. Skierka J. M. Blommel J. H. Moore B. E. VanCuyk D. L. Bruflat J. K.. (2016). Preemptive pharmacogenomic testing for precision medicine: a comprehensive analysis of five actionable pharmacogenomic genes using next-generation DNA sequencing and a customized CYP2D6 genotyping cascade. J. Mol. Diagn. 18, 438–445. 10.1016/j.jmoldx.2016.01.003 26947514 PMC4851731

[B22] Joosten E. A. DeFuentes-Merillas L. de Weert G. H. Sensky T. van der Staak C. P. de Jong C. A. (2008). Systematic review of the effects of shared decision-making on patient satisfaction, treatment adherence and health status. Psychother. Psychosom. 77, 219–226. 10.1159/000126073 18418028

[B23] Kim K. M. Murray M. D. Tu W. Robarge J. Ding Y. Brater D. C.. (2012). Pharmacogenetics and healthcare outcomes in patients with chronic heart failure. Eur. J. Clin. Pharmacol. 68, 1483–1491. 10.1007/s00228-012-1280-z 22543981

[B24] Kirchheiner J. Schmidt H. Tzvetkov M. Keulen J. T. Lotsch J. Roots I.. (2007). Pharmacokinetics of codeine and its metabolite morphine in ultra-rapid metabolizers due to CYP2D6 duplication. Pharmacogenomics J. 7, 257–265. 10.1038/sj.tpj.6500406 16819548

[B25] Lazaridis K. N. (2019). PACE forward-making pharmacogenomics testing available for real-life clinical utility. Clin. Pharmacol. Ther. 105, 42–44. 10.1002/cpt.1274 30520030

[B26] Leendertse A. J. Egberts A. C. Stoker L. J. van den Bemt P. M. (2008). Frequency of and risk factors for preventable medication-related hospital admissions in the Netherlands. Arch. Intern. Med. 168, 1890–1896. 10.1001/archinternmed.2008.3 18809816

[B27] Liberati A. Altman D. G. Tetzlaff J. Mulrow C. GÃtzsche P. C. Ioannidis J. P. A.. (2009). The PRISMA statement for reporting systematic reviews and meta-analyses of studies that evaluate healthcare interventions: explanation and elaboration. BMJ 339:b2700. 10.1136/bmj.b2700 19622552 PMC2714672

[B28] Lorizio W. Rugo H. Beattie M. S. Tchu S. Melese T. Melisko M.. (2011). Pharmacogenetic testing affects choice of therapy among women considering tamoxifen treatment. Genome Med. 3:64. 10.1186/gm280 21970596 PMC3239226

[B29] Luzum J. A. Pakyz R. E. Elsey A. R. Haidar C. E. Peterson J. F. Whirl-Carrillo M.. (2017). The pharmacogenomics research network translational pharmacogenetics program: outcomes and metrics of pharmacogenetic implementations across diverse healthcare systems. Clin. Pharmacol. Ther. 102, 502–510. 10.1002/cpt.630 28090649 PMC5511786

[B30] Mai Y. Mitropoulou C. Papadopoulou X. E. Vozikis A. Cooper D. N. van Schaik R. H.. (2014). Critical appraisal of the views of healthcare professionals with respect to pharmacogenomics and personalized medicine in Greece. Per. Med. 11, 15–26. 10.2217/pme.13.92 29751393

[B31] Manson L. E. van der Wouden C. H. Swen J. J. Guchelaar H. J. (2017). The ubiquitous pharmacogenomics consortium: making effective treatment optimization accessible to every European citizen. Pharmacogenomics 18, 1041–1045. 10.2217/pgs-2017-0093 28685652

[B32] McInnes G. Lavertu A. Sangkuhl K. Klein T. E. Whirl-Carrillo M. Altman R. B. (2021). Pharmacogenetics at scale: an analysis of the UK Biobank. Clin. Pharmacol. Ther. 109, 1528–1537. 10.1002/cpt.2122 33237584 PMC8144239

[B33] Mitropoulou C. Litinski V. Kabakchiev B. Rogers S. P. Patrinos G. (2020). PARC report: health outcomes and value of personalized medicine interventions: impact on patient care. Pharmacogenomics 21, 797–807. 10.2217/pgs-2019-0194 32635813

[B34] Mostafa S. Kirkpatrick C. M. J. Byron K. Sheffield L. (2019). An analysis of allele, genotype and phenotype frequencies, actionable pharmacogenomic (PGx) variants and phenoconversion in 5408 Australian patients genotyped for CYP2D6, CYP2C19, CYP2C9 and VKORC1 genes. J. Neural. Transm. 126, 5–18. 10.1007/s00702-018-1922-0 30191366

[B35] Papastergiou J. Tolios P. Li W. Li J. (2017). The innovative canadian pharmacogenomic screening initiative in community pharmacy (ICANPIC) study. J. Am. Pharm. Assoc. 57, 624–629. 10.1016/j.japh.2017.05.006 28689706

[B36] Patel J. N. Mueller M. K. Guffey W. J. Stegman J. (2020). Drug prescribing and outcomes after pharmacogenomic testing in a developmental and behavioral health pediatric clinic. J. Dev. Behav. Pediatr. 41, 65–70. 10.1097/DBP.0000000000000746 31688658

[B37] Pedrós C. Quintana B. Rebolledo M. Porta N. Vallano A. Arnau J. M. (2014). Prevalence, risk factors and main features of adverse drug reactions leading to hospital admission. Eur. J. Clin. Pharmacol. 70, 361–367. 10.1007/s00228-013-1630-5 24362489

[B38] Perlis R. H. Dowd D. Fava M. Lencz T. Krause D. S. (2020). Randomized, controlled, participant- and rater-blind trial of pharmacogenomic test-guided treatment versus treatment as usual for major depressive disorder. Depress. Anxiety 37, 834–841. 10.1002/da.23029 32383277

[B39] Perlis R. H. Mehta R. Edwards A. M. Tiwari A. Imbens G. W. (2018). Pharmacogenetic testing among patients with mood and anxiety disorders is associated with decreased utilization and cost: A propensity-score matched study. Depress Anxiety 35, 946–952. 10.1002/da.22742 29734486

[B40] Popay J. Roberts H. Sowden A. Petticrew M. Arai L. Rodgers M.. (2006). Guidance on the Conduct of Narrative Synthesis in Systematic Reviews: A Product From the ESRC Methods Programme. Lancaster, PA: University of Lancaster.

[B41] Povsic T. J. Ohman E. M. Roe M. T. White J. Rockhold F. W. Montalescot G.. (2019). P2Y12 inhibitor switching in response to routine notification of CYP2C19 clopidogrel metabolizer status following acute coronary syndromes. JAMA Cardiol. 4, 680–684. 10.1001/jamacardio.2019.1510 31141104 PMC6547088

[B42] Relling M. V. Evans W. E. (2015). Pharmacogenomics in the clinic. Nature 526, 343–350. 10.1038/nature15817 26469045 PMC4711261

[B43] Ruaño G. Robinson S. Holford T. Mehendru R. Baker S. Tortora J.. (2020). Results of the CYP-GUIDES randomized controlled trial: Total cohort and primary endpoints. Contemp. Clin. Trials 89:105910. 10.1016/j.cct.2019.105910 31838256

[B44] Ruddy K. J. Desantis S. D. Gelman R. S. Wu A. H. Punglia R. S. Mayer E. L.. (2013). Personalized medicine in breast cancer: tamoxifen, endoxifen, and CYP2D6 in clinical practice. Breast Cancer Res. Treat. 141, 421–427. 10.1007/s10549-013-2700-1 24062210

[B45] Sharma M. Kantorovich S. Lee C. Anand N. Blanchard J. Fung E. T.. (2017). An observational study of the impact of genetic testing for pain perception in the clinical management of chronic non-cancer pain. J. Psychiatr. Res. 89, 65–72. 10.1016/j.jpsychires.2017.01.015 28182962

[B46] Sharp C. N. Linder M. W. Valdes R. (2019). Polypharmacy: a healthcare conundrum with a pharmacogenetic solution. Crit. Rev. Clin. Lab. Sci. 2, 1–20. 10.1080/10408363.2019.1678568 31680605 PMC7195220

[B47] Silva D. D. (2012). Evidence: Helping People Share Decision Making. London: The Health Foundation.

[B48] Thase M. E. Parikh S. V. Rothschild A. J. Dunlop B. W. DeBattista C. Conway C. R.. (2019). Impact of pharmacogenomics on clinical outcomes for patients taking medications with gene-drug interactions in a randomized controlled trial. J. Clin. Psychiatry 80:19m12910. 10.4088/JCP.19m12910 31721487

[B49] Turner R. M. Newman W. G. Bramon E. McNamee C. J. Wong W. L. Misbah S.. (2020). Pharmacogenomics in the UK National Health Service: opportunities and challenges. Pharmacogenomics 21, 1237–1246. 10.2217/pgs-2020-0091 33118435

[B50] Tuteja S. Glick H. Matthai W. Nachamkin I. Nathan A. Monono K.. (2020). Prospective *CYP2C19* genotyping to guide antiplatelet therapy following percutaneous coronary intervention: a pragmatic randomized clinical trial. Circ. Genom. Precis. Med. 13:e002640. 10.1161/CIRCGEN.119.002640 31928229

[B51] Van Driest S. L. Shi Y. Bowton E. A. Schildcrout J. S. Peterson J. F. Pulley J.. (2014). Clinically actionable genotypes among 10,000 patients with preemptive pharmacogenomic testing. Clin. Pharmacol. Ther. 95, 423–431. 10.1038/clpt.2013.229 24253661 PMC3961508

[B52] Vassy J. L. Brunette C. A. Majahalme N. Advani S. MacMullen L. Hau C.. (2018). The Integrating Pharmacogenetics in Clinical Care (I-PICC) Study: protocol for a point-of-care randomized controlled trial of statin pharmacogenetics in primary care. Contemp. Clin. Trials 75, 40–50. 10.1016/j.cct.2018.10.010 30367991 PMC8119226

[B53] Viechtbauer W. Cheung M. W. (2010). Outlier and influence diagnostics for meta-analysis. Res. Synth. Methods 1, 112–125. 10.1002/jrsm.11 26061377

[B54] Wei C. Y. Lee M. T. Chen Y. T. (2012). Pharmacogenomics of adverse drug reactions: implementing personalized medicine. Hum. Mol. Genet. 21, R58–R65. 10.1093/hmg/dds341 22907657

[B55] Winner J. G. Carhart J. M. Altar C. A. Allen J. D. Dechairo B. M. (2013). A prospective, randomized, double-blind study assessing the clinical impact of integrated pharmacogenomic testing for major depressive disorder. Discov. Med. 6, 219–227. 24229738

