# Supplementary material for: An Analysis of Pharmacogenomic-Guided Pathways and Their Effect on Medication Changes and Hospital Admissions: A Systematic Review and Meta-Analysis
Source: Front Genet. 2021 Jul 30;12:698148. doi: 10.3389/fgene.2021.698148 (PMC8362615; doi:10.3389/fgene.2021.698148)
Supplement: Supplementary file 1 [file Data_Sheet_1.docx]

Supplementary Material

**Supplementary Table 1 Quality Assessment Tools used for all reviewed papers on the hospitalisation theme: CASP checklist**

| CASP tool criteria | Brixner et al. (2016) |  | Epstein et al. (2010) | Perlis et al. (2018) |  |
| --- | --- | --- | --- | --- | --- |
| Observational studies |  |  |  |  |  |
| Are the results of the study valid? (Relates to validity) | | | | | |
| 1. Did the study address a clearly focused issue? (Cohort or Case-control study) | ✓ |  | ✓ | **✓** |  |
| 1. Was the cohort recruited in an acceptable way? (Cohort study) OR   **Did the authors use an appropriate method to answer their question? (Case control study)** | ✓ |  | ✓ | **✓** |  |
| 1. Was the exposure accurately measured to minimise bias? (Cohort study)   OR  **Were the cases recruited in an acceptable way? (Case control study)** | ✓ |  | ✓ | **✓** |  |
| 1. Was the outcome accurately measured to minimise bias? (Cohort study)   OR  **Were the controls selected in an acceptable way? (Case control study)** | ✓ |  | ✓ | **✓** |  |
| 1. A. Have the authors identified all important confounding factors?   B. Have they taken account of the confounding factors in the design and/or analysis? (Cohort study)  OR  **Was the exposure accurately measured to minimise bias? (Case control study)** | Χ      ✓ |  | Χ      ✓ | **✓** |  |
| 1. A. Was the follow up of subjects complete enough?   B. Was the follow up of subjects long enough? (Cohort study)  OR   1. **Aside from the experimental intervention, were the groups treated equally? (Case control study)** 2. **Have the authors taken account of the potential confounding factors in the design and/or in their analysis? (Case control study)** | ✓    Χ |  | ✓    ✓ | **✓**        **✓** |  |
| What are the results? (Relates to trustworthiness of results) |  |  |  |  |  |
| 1. What are the results of this study? (Cohort study) OR   **How large was the treatment effect? (Case control study)** | See figure 5 and table 3 |  | See figure 5 and table 3 | **See figure 5 and table 3** |  |
| 1. How precise are the results? (Cohort study)   OR  **How precise was the estimate of the treatment effect? (Case control study)** | Confidence intervals are p<0.05 |  | Confidence intervals are p<0.05 | Confidence intervals are p<0.05 |  |
| 1. Do you believe the results? (Both study types) | ✓ |  | ✓ | **✓** |  |
| Randomised Controlled Studies |  | Elliott et al. (2017) |  |  | Ruaño et al. (2020) |
| Is the basic study design valid for a randomised controlled trial? | | | | | |
| 1. Did the study address a clearly focused research question? |  | ✓ |  |  | ✓ |
| 1. Was the assignment of participants to interventions randomised? |  | ✓ |  |  | ✓ |
| 1. Were all participants who entered the study accounted for its conclusions? |  | ✓ |  |  | ✓ |
| Was the study methodologically sound? |  |  |  |  |  |
| 1. -Were the participants ‘blind’ to intervention they were given?   -Were the investigators ‘blind’ to the intervention they were giving to participants?    -Were the people assessing/analysing outcome/s ‘blinded’? |  | Χ    Χ      Χ |  |  | ✓    Χ      Χ |
| 1. Were the study groups similar at the start of the randomised controlled trial? |  | ✓ |  |  | ✓ |
| 1. Apart from the experimental intervention, did each study group receive the same level of care (that is, were they treated equally)? |  | ✓ |  |  | ✓ |
| What are the results? (Relates to trustworthiness of results) |  |  |  |  |  |
| 1. Were the effects of the intervention reported comprehensively? |  | ✓ |  |  | Χ |
| 1. Was the precision of the estimate of the intervention or treatment effect reported? |  | ✓ |  |  | Χ |
| 1. Do the benefits of the experimental intervention outweigh the harms and costs? |  | ✓ |  |  | - |

**Supplementary Table 2 Quality Assessment Tools used for all reviewed papers on the medication change theme**

| CASP tool criteria | Brixner et al. (2016) | Hall-Flavin et al. (2013), US |  |  |  |
| --- | --- | --- | --- | --- | --- |
| Observational cohort study |  |  |  |  |  |
| Are the results of the study valid? (Relates to validity) | | | | | |
| 1. Did the study address a clearly focused issue? | ✓ | ✓ |  |  |  |
| 1. Was the cohort recruited in an acceptable way? | ✓ | ✓ |  |  |  |
| 1. Was the exposure accurately measured to minimise bias? | ✓ | ✓ |  |  |  |
| 1. Was the outcome accurately measured to minimise bias? | ✓ | ✓ |  |  |  |
| 1. A. Have the authors identified all important confounding factors?     B. Have they taken account of the confounding factors in the design and/or analysis? | Χ  ✓ | Χ    ✓ |  |  |  |
| 1. A. Was the follow up of subjects complete enough?     B. Was the follow up of subjects long enough? | ✓    Χ | ✓    ✓ |  |  |  |
| What are the results? (Relates to trustworthiness of results) |  |  |  |  |  |
| 1. What are the results of this study? | See figure 4 | See figure 4 |  |  |  |
| 1. How precise are the results? | Confidence intervals are p<0.05 | Confidence intervals are p<0.0001 |  |  |  |
| 1. Do you believe the results? | ✓ | ✓ |  |  |  |
| Randomised Controlled Studies |  |  | Thase et al. (2019), US | Tuteja et al. (2020), US | Winner et al. (2013), US |
| Is the basic study design valid for a randomised controlled trial? | | | | | |
| 1. Did the study address a clearly focused research question? |  |  | ✓ | ✓ | ✓ |
| 1. Was the assignment of participants to interventions randomised? |  |  | ✓ | ✓ | ✓ |
| 1. Were all participants who entered the study accounted for its conclusions? |  |  | ✓ | ✓ | ✓ |
| Was the study methodologically sound? |  |  |  |  |  |
| -Were the participants ‘blind’ to intervention they were given?    -Were the investigators ‘blind’ to the intervention they were giving to participants?    -Were the people assessing/analysing outcome/s ‘blinded’? |  |  | ✓      ✓  ✓ | Χ        Χ      Χ | ✓        Χ      ✓ |
| 1. Were the study groups similar at the start of the randomised controlled trial? |  |  | ✓ | ✓ | ✓ |
| 1. Apart from the experimental intervention, did each study group receive the same level of care (that is, were they treated equally)? |  |  | ✓ | ✓ | ✓ |
| What are the results? (Relates to trustworthiness of results) |  |  |  |  |  |
| 1. Were the effects of the intervention reported comprehensively? |  |  | ✓ | ✓ | ✓ |
| 1. Was the precision of the estimate of the intervention or treatment effect reported? |  |  | ✓ | ✓ | ✓ |
| 1. Do the benefits of the experimental intervention outweigh the harms and costs? |  |  | ✓ | ✓ | ✓ |


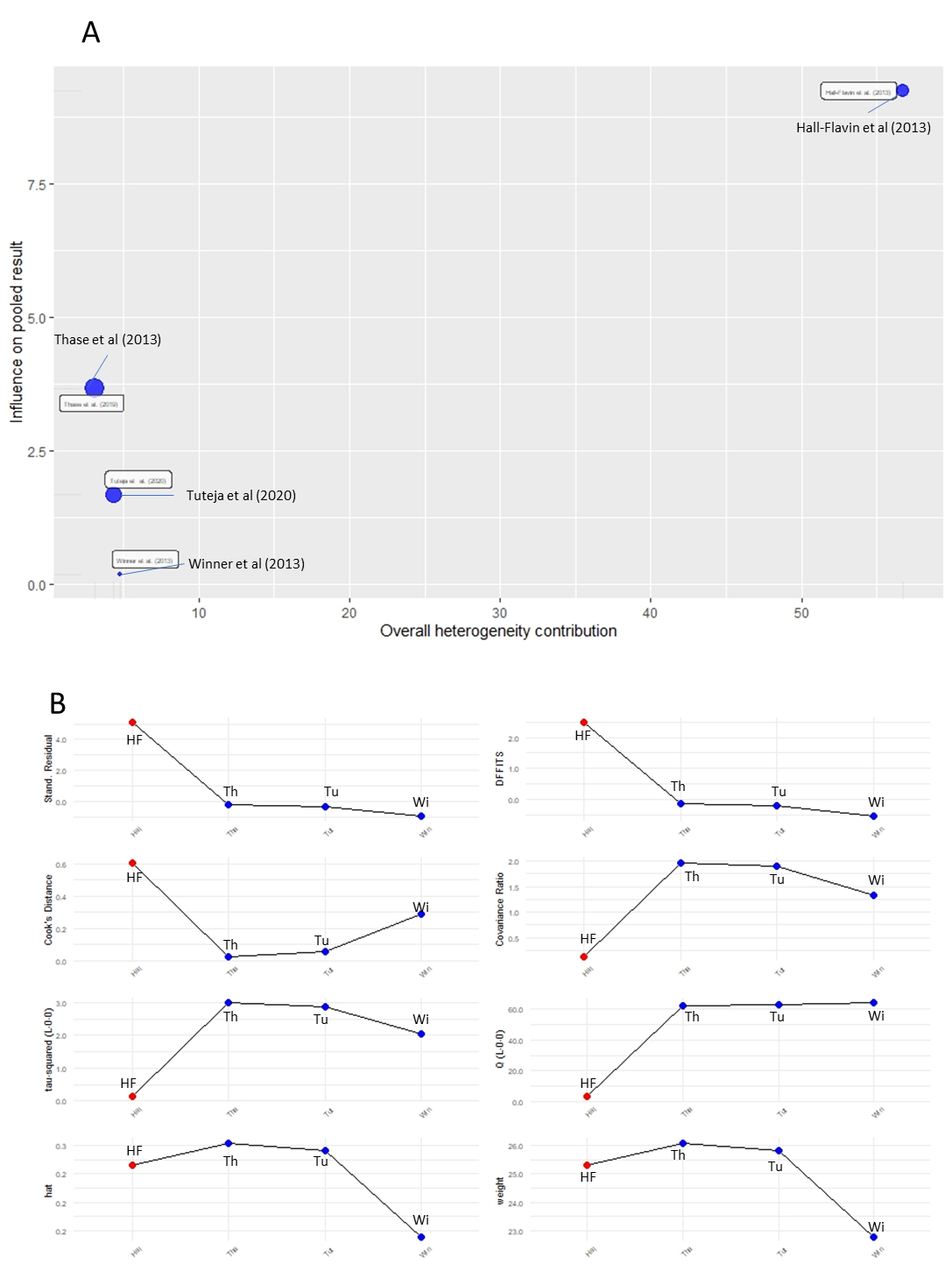


*Supplementary figure 1: Medication changes (a) Baujat plot (b) Influence analyses. Included studies and their abbreviations:* ***HF*** *is Hall-Flavin et al.(2013),* ***Th*** *is Thase et al.(2019),* ***Tu*** *is Tuteja et al.(2020) and* ***Wi*** *is Winner et al. (2013).*


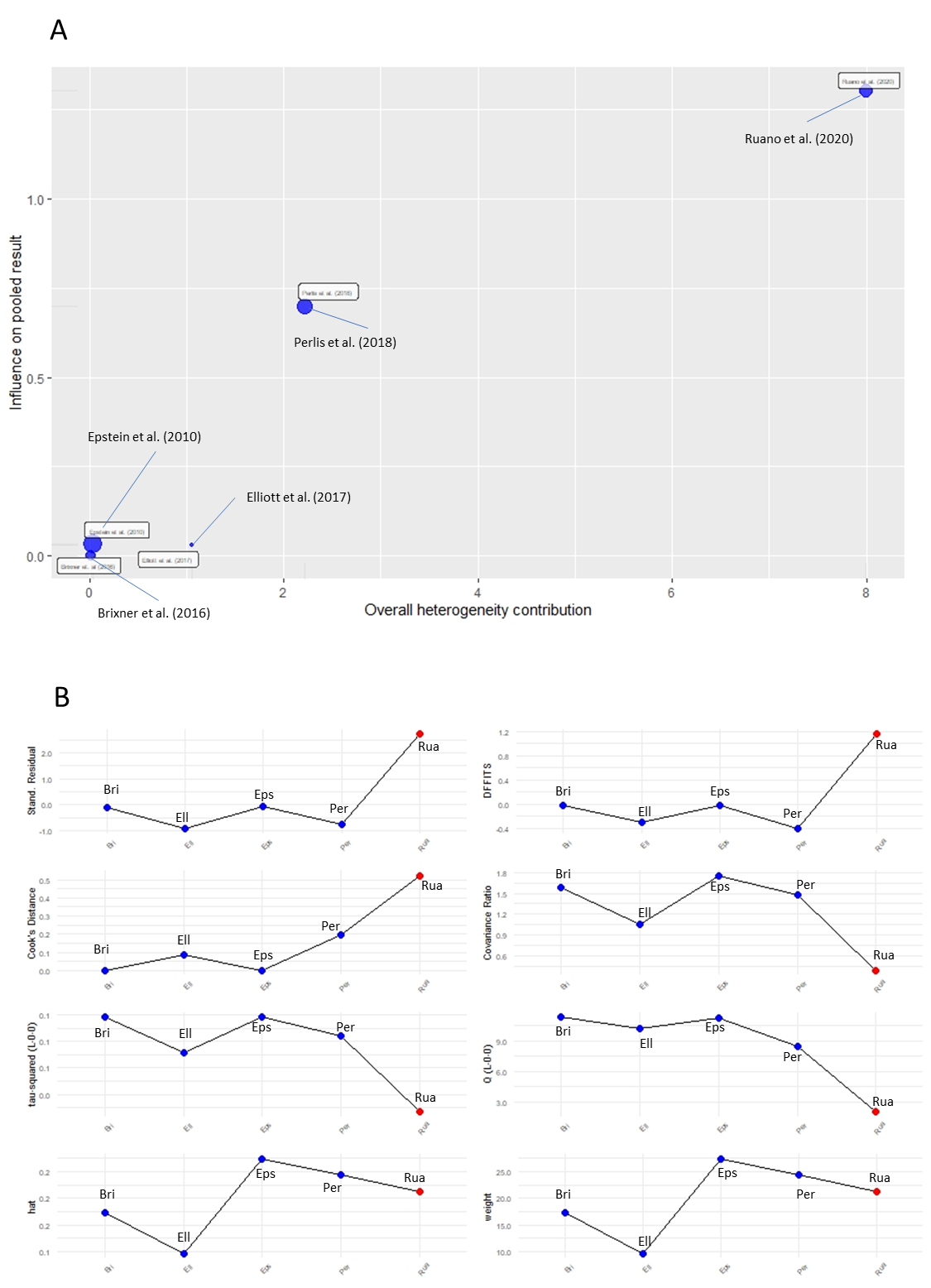


*Supplementary Figure 2: Hospitalisation (A) Baujat plot (B) Influence analyses. Included studies and their abbreviations:* ***Bri*** *is Brixner et al. (2016),* ***Ell*** *is Elliott et al. (2017),* ***Eps*** *is Epstein et al.(2010),* ***Per*** *is Perlis et al.(2018) and* ***Rua*** *is Ruano et al.(2020)*
